# Oculomics analysis in multiple sclerosis: Current ophthalmic clinical and imaging biomarkers

**DOI:** 10.1038/s41433-024-03132-y

**Published:** 2024-06-10

**Authors:** Alex Suh, Gilad Hampel, Aditya Vinjamuri, Joshua Ong, Sharif Amit Kamran, Ethan Waisberg, Phani Paladugu, Nasif Zaman, Prithul Sarker, Alireza Tavakkoli, Andrew G. Lee

**Affiliations:** 1 Tulane University School of Medicine, New Orleans, LA USA; 2 Michigan Medicine, University of Michigan, Ann Arbor, MI USA; 3 Human-Machine Perception Laboratory, Department of Computer Science and Engineering, University of Nevada, Reno, Reno, NV USA; 4 University College Dublin School of Medicine, Belfield, Dublin Ireland; 5 Brigham and Women’s Hospital, Harvard Medical School, Boston, MA USA; 6 Sidney Kimmel Medical College, Thomas Jefferson University, Philadelphia, PA USA; 7 Center for Space Medicine, Baylor College of Medicine, Houston, TX USA; 8 Department of Ophthalmology, Blanton Eye Institute, Houston Methodist Hospital, Houston, TX USA; 9 The Houston Methodist Research Institute, Houston Methodist Hospital, Houston, TX USA; 10 Departments of Ophthalmology, Neurology, and Neurosurgery, Weill Cornell Medicine, New York, NY USA; 11 Department of Ophthalmology, University of Texas Medical Branch, Galveston, TX USA; 12 University of Texas MD Anderson Cancer Center, Houston, TX USA; 13 Texas A&M College of Medicine, Galveston, TX USA; 14 Department of Ophthalmology, The University of Iowa Hospitals and Clinics, Iowa City, IA USA

**Keywords:** Prognostic markers, Education

## Abstract

Multiple Sclerosis (MS) is a chronic autoimmune demyelinating disease of the central nervous system (CNS) characterized by inflammation, demyelination, and axonal damage. Early recognition and treatment are important for preventing or minimizing the long-term effects of the disease. Current gold standard modalities of diagnosis (e.g., CSF and MRI) are invasive and expensive in nature, warranting alternative methods of detection and screening. Oculomics, the interdisciplinary combination of ophthalmology, genetics, and bioinformatics to study the molecular basis of eye diseases, has seen rapid development through various technologies that detect structural, functional, and visual changes in the eye. Ophthalmic biomarkers (e.g., tear composition, retinal nerve fibre layer thickness, saccadic eye movements) are emerging as promising tools for evaluating MS progression. The eye’s structural and embryological similarity to the brain makes it a potentially suitable assessment of neurological and microvascular changes in CNS. In the advent of more powerful machine learning algorithms, oculomics screening modalities such as optical coherence tomography (OCT), eye tracking, and protein analysis become more effective tools aiding in MS diagnosis. Artificial intelligence can analyse larger and more diverse data sets to potentially discover new parameters of pathology for efficiently diagnosing MS before symptom onset. While there is no known cure for MS, the integration of oculomics with current modalities of diagnosis creates a promising future for developing more sensitive, non-invasive, and cost-effective approaches to MS detection and diagnosis.

## Abstract (zh)

多发性硬化(MS)是一种以慢性自身免疫性中枢神经系统(CNS)受累为特征的脱髓鞘疾病, 以炎症、脱髓鞘和轴突损伤为临床特征。早期识别和治疗对于预防或最大限度地减少疾病的长期损伤至关重要。目前诊断的金标准 (如脑脊液检测和核磁共振)具有侵入性和价格昂贵等缺点, 需探索检测和筛查的替代方法。眼部组学是眼科学、遗传学和生物信息学的交叉学科, 旨在研究眼病的分子基础, 通过检测眼的结构、功能和视觉变化的各项技术的革新, 得到了快速发展。眼科生物标志物(如泪液、视网膜神经纤维层厚度、眼球扫视运动)将成为评估MS进展的有前景的工具。眼与脑在结构和胚胎学上的相似之处为其评估中枢神经系统的神经和微血管变化提供了潜在可能。随着更强大的机器学习算法的出现, 眼部组学筛查方式, 如相干光断层扫描技术(OCT)、眼跟踪和蛋白质分析, 成为MS辅助诊断的更有效工具。人工智能可分析更大、更多样化的数据集, 可能发现新的病理学参数, 以便在症状出现前有效诊断MS。虽然MS的治疗方法未知, 但眼部组学与当前诊断方法的结合有望在未来开发更敏感、无创和更具成本效益的MS检测和诊断方法。

## Introduction

Multiple Sclerosis (MS) is a chronic autoimmune demyelinating disease of the central nervous system (CNS) characterized by inflammation, demyelination, and axonal damage. The debilitating effects of MS are experienced by 2.8 million people worldwide, with a greater prevalence in regions further from the equator [1]. MS has a variable clinic course, with different identified clinical subtypes including relapsing-remitting MS (RRMS), secondary progressive MS (SPMS), primary progressive MS (PPMS), and relapsing-progressive MS (RPMS) [2]. Early recognition and treatment are important for preventing or minimizing the long-term effects of the disease. Emerging research has focused on the development of new diagnostic tools, biomarkers, and mechanistic treatments for MS.

The diagnosis of MS has historically been based on clinical observations of two or more objective clinical demyelinating events separated in space and time. Radiographic visualization of characteristic demyelinating white matter lesions in the CNS using cranial magnetic resonance imaging (MRI) and cerebrospinal fluid (CSF) analysis confirming oligoclonal bands were the commonly accepted tools to support a clinical suspicion of MS [3]. The recent emergence of potential reliable biomarkers has opened new doors for earlier detection, more accurate diagnosis, and more precise monitoring of disease progression in MS. Oculomics is the study of ocular manifestations and their relationship to systemic diseases. The retina and optic nerve, for example, provide a direct extension of the brain, allowing non-invasive real-time depiction of the function and microvascular structure of the CNS [4]. In the context of MS, optic neuritis is a common initial clinical presentations of MS, providing a potential early opportunity to screen for and diagnose MS. However, oculomics extends beyond the retina and optic nerve, and new research has focused on novel biomarkers in ocular fluids as well as eye-tracking movements.

**Fig. 1:**
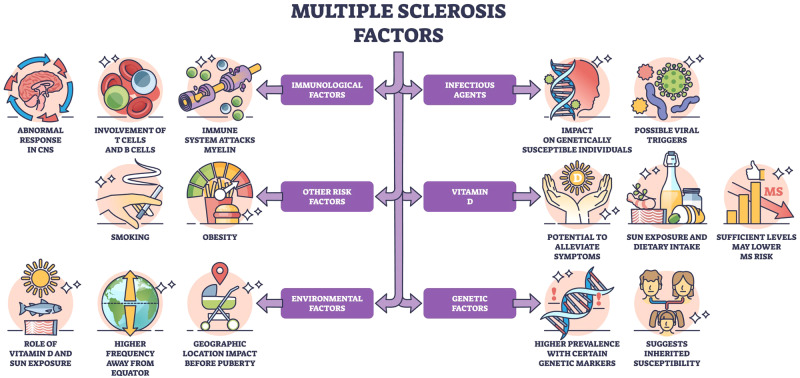
The Risk factors for Multiple Sclerosis. MS is considered a multifactorial disease with several associated risk factors (e.g., genetics, immunologic factors, insufficient vitamin D, environment). The underlying cause of pathological changes (e.g., white matter lesions, ocular findings) in MS is still unknown. Several hypotheses propose that chronic inflammation, degeneration, and demyelination of axons are mediated by T-lymphocytes mistakenly targeting components of the CNS as if they were foreign pathogens [1]. Activated T cells cross the blood-brain barrier and recognize self-antigen presented by antigen-presenting cells (APCs) within the CNS. Subsequently, proinflammatory cytokines (e.g., tumour necrosis factor-alpha, interferon-gamma) may be triggered to promote the recruitment of additional immune cells and contribute to the positive feedback loop inflammatory process. Image is under Creative Commons Attribution-NonCommercial-NoDerivatives 4.0 International (CC BY-NC-ND 4.0) License (https://creativecommons.org/licenses/by-nc-nd/4.0/legalcode).

Earlier detection of MS provides an opportunity for more timely initiation of disease-modifying therapies (DMTs) for MS. DMTs have been shown to delay disease progression, improve long-term outcomes, and reduce annual relapse rates by 29% to 68% compared with placebos [5]. Since MS overlaps with other inflammatory and autoimmune demyelinating conditions which may present with similar symptoms and signs, early identification may be critical to avoid unnecessary investigations and appropriate or ineffective treatments. In the era of machine learning and artificial intelligence (AI), the screening and diagnostic process of oculomics in MS has increased potential to improve the accuracy, efficiency, and objectivity of diagnosis. Machine learning algorithms can analyse large datasets of ocular imaging and biomarker data to identify patterns and generate predictive models [6]. These training models can be based on known MS cases and may allow the creation of diagnostic tools for healthcare professionals in accurately interpreting clinical, laboratory, and imaging results, flagging potential abnormalities, identifying risk factors, and providing decision support for diagnosis (Fig. 1).

Current methods of early MS detection involve a combination of clinical assessment, neuroimaging, and laboratory (e.g., CSF) investigations. Clinical evaluation of medical history, neurological examination (e.g., hyperreflexia, hyporeflexia, vision problems, numbness, tingling), and symptom assessment of mood, fatigue, musculoskeletal pain may provide indication of MS [7]. Biomarkers for MS include MRI of lesions in the brain and spinal cord, CSF analysis of specific proteins (e.g., IgG and albumin), oligoclonal bands, and serum biomarkers (e.g., neurofilament light chain, soluble CD27) [8]. Some studies have already demonstrated the potential of machine learning and AI in the MRI-based subtyping of MS [9]. This allows providers to administer more subtype-specific treatment plans, which increases the likelihood of treatment response and patient satisfaction [10].

DMTs, such as interferons, monoclonal antibodies, and the latest oral or infusion therapies, aim to reduce inflammation, delay disease progression, and minimize relapses [11]. While there is no known cure for MS, the integration of oculomics with current modalities of diagnosis creates a promising future of developing more sensitive, non-invasive, and cost-effective approaches to MS detection and diagnosis. Thus, healthcare providers may implement more appropriate treatment strategies to mitigate disability and optimize long-term outcomes for MS patients.

## Methods

A systematic review was performed following PRISMA 2020 (Preferred Reporting Items for Systematic Reviews and Meta-Analyses) guidelines. From June to December 2023, PubMed and Google Scholar were searched to identify studies related to Oculomics Analysis of patients with MS. Basic science papers exploring MS, randomized controlled trials, systematic reviews, and meta-analyses published between 1970-present, and had data from male and/or female patients between the ages of 8-85 years old were included in the results. Only those papers published in full-text were included. Case reports, letters to the editor, conference abstracts, and animal studies were excluded.

From the retrieved articles, relevant articles based on eligibility criteria and the presence of keywords in the title or abstract were selected. Inclusion criteria were (1) studies conducted between 1970-present, randomized control trials, systematic reviews, meta-analyses, and basic science publications related to the clinical population of multiple sclerosis (2) use of relevant techniques (e.g., optical coherence tomography (OCT), OCT angiography (OCTA), electroretinography (ERG)); and (3) investigating two groups of participants (healthy matched controls and patients with disease). Two authors independently screened the titles and abstracts of all studies that incorporated keywords or were thematically related to the oculomics of MS. Independently, they retrieved and reviewed the full-text articles that met inclusion criteria. Any disparities were resolved through discussion, and if consensus could not be achieved, a senior reviewer was consulted.

### Study selection

**Fig. 2:**
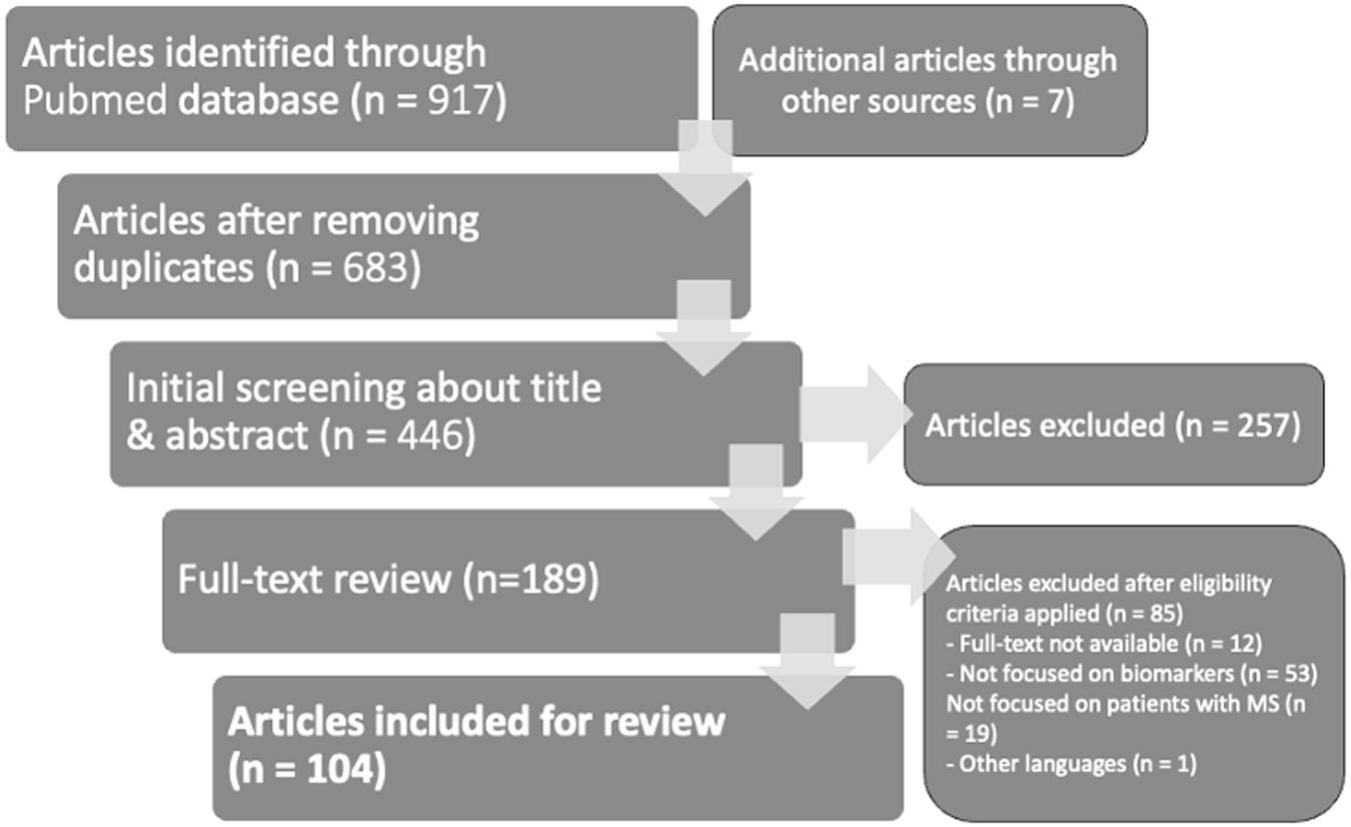
PRISMA Flow Diagram of Identification, Screening, Eligibility, and Inclusion process applied during the systematic review. PRISMA Flow Diagram showing the systematic review process: stages of identification, screening, eligibility, and inclusion, resulting in 104 included articles.

The studies were selected according to the PRISMA flow chart outlined in Fig. 2. The original search through two databases provided 917 articles. After removing 234 duplicates and articles which did not pertain to oculomics of MS, 683 articles were retained. Following an initial screening process, 257 articles were excluded based on predetermined exclusion criteria. The remaining 426 articles were evaluated if they met the inclusion criteria of which 237 did not. The full text of 189 articles was retrieved and reviewed based on the predetermined inclusion criteria. During the full-text review, 85 full-text articles were excluded, resulting in their disqualification, ending with the total selection of 104 articles.

### The retina as a potential biomarker

#### Optical Coherence Tomography (OCT)

##### Retinal Nerve Fibre Layer (RNFL)

A post-mortem analysis of the retina and optic nerve has revealed changes in almost all of the MS patients, regardless of experienced optic neuritis [2, 3]. OCT is a non-invasive ocular imaging technique that utilizes light waves to generate high-resolution, cross-sectional images of ocular structures. The non-invasive, quick, and affordable characteristics make it a prime method for assessing axonal and neuronal degeneration in MS [12]. OCT studies have demonstrated a significant reduction in retinal nerve fibre layer (RNFL) and ganglion cell layer (GCL) thickness in patients with MS (with or without optic neuritis) [13, 14]. RNFL thickness of affected eyes has been reported to be reduced by an average of 46% (*P* < 0.01). When compared to the unaffected eye of the patients, the affected eye had an average RNFL reduction of 28% (*P* < 0.01). When comparing the unaffected eyes to the control eyes, there was an average of 26% reduction in RNFL (*P* < 0.01). In MS patients with acute optic neuritis, a 10–40 μm loss of RNFL thickness was observed in approximately 75% within three to six months of the initial inflammatory event [3]. Moreover, it is worth noting that peripapillary RNFL thickness shows a decrease not only in MS patients with optic neuritis but also in those without any reported ocular complaints. The continuous degenerative injury to the anterior visual pathways may manifest, even without a clinically diagnosed acute optic neuritis, highlighting the essential role of comprehensive monitoring in the management of MS [4]. However, it is crucial to note that continuous monitoring may not be deemed necessary in all cases, and a balanced approach to surveillance can be considered based on individual patient characteristics and clinical indicators.

RNFL thinning is a predictor of visual loss (confirmed through perimetry or low-contrast letter acuity), further emphasizing OCT’s utility as a non-invasive measure for monitoring disease course [5]. Reduction in RNFL, however, is nonspecific for MS and can be observed in other disorders (e.g., neuromyelitis optic spectrum disorder, Susac Syndrome) [6]. Therefore symptom context and distribution of thinning is needed to make a differential diagnosis [15]. For example, in patients with neuromyelitis optica spectrum disorder, RNFL loss tended to be more diffusely distributed and severe, whereas MS optic neuritis (MSON) patients had RNFL loss concentrated in the temporal quadrant [16]. Similarly, Susac syndrome patients demonstrate a wider distribution of thinning in the retinal and macular areas, whereas MS patients had more distinct sectorial loss in the macular regions [17, 18]. With a larger database of specific locations and changes in the RNFL, OCT may soon be a more useful tool in the differential diagnosis of diseases with similar retinal and macular changes as MS. In this context, enhanced diagnostic criteria for optic neuritis and its subgroups could further improve the precision of differential diagnoses and guide treatment decisions based on the observed patterns of RNFL thinning and optic neuritis.

OCT emerges as a powerful and non-invasive tool for assessing axonal and neuronal degeneration in MS. Key findings in RNFL analysis reveal significant reductions in thickness in MS patients, both with and without optic neuritis, emphasizing its potential as a comprehensive biomarker for monitoring disease progression. However, a balanced approach to surveillance is advised, considering individual patient characteristics and clinical indicators. Future directions for RNFL as a biomarker in MS involve refining its specificity for differential diagnoses. While RNFL reduction is nonspecific to MS, the distinctive patterns observed in different disorders (e.g. neuromyelitis optica spectrum disorder, Susac syndrome) highlight the potential for OCT to contribute significantly to differential diagnoses. As databases expand to include specific locations and changes in RNFL, OCT may become an increasingly valuable tool for precise disease differentiation. Enhanced diagnostic criteria for optic neuritis and its subgroups can further improve the precision of differential diagnoses, guiding treatment decisions based on observed patterns of RNFL thinning and optic neuritis. The ongoing evolution of OCT technology and its application in MS holds promising avenues for enhancing diagnostic accuracy and refining personalized treatment strategies.

##### Ganglion Cell Layer and Inner Plexiform Layer (GCIPL)

**Fig. 3:**
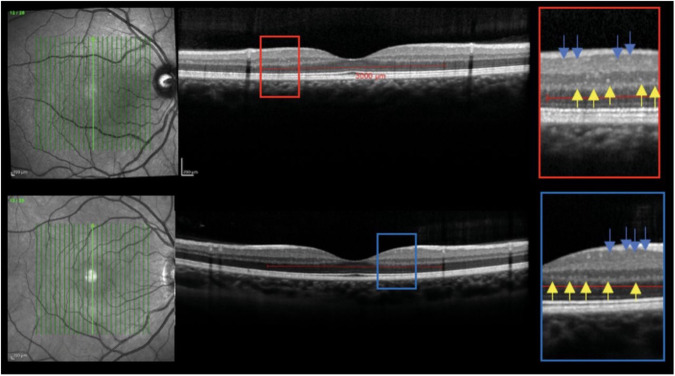
Optical coherence tomography (OCT) and hyperreflective foci in a patient with relapsing-remitting multiple sclerosis (top OCT) and healthy control (bottom OCT). The foci in the inner nuclear layer are indicated by yellow arrows, and the foci in the ganglion cell and inner plexiform layer are indicated by the blue arrows. Pengo et al. [19]. observed an association of hyperreflective foci, an indicator of activated and proliferating retinal microglial, on OCT in relapsing-remitting multiple sclerosis compared to healthy controls. Reprinted with permission from Pengo et al. [19]. Retinal Hyperreflecting Foci Associated With Cortical Pathology in Multiple Sclerosis. Neurol Neuroimmunol Neuroinflamm. 2022 May 23;9(4):e1180 under Creative Commons Attribution-NonCommercial-NoDerivatives 4.0 International (CC BY-NC-ND 4.0) License (https://creativecommons.org/licenses/by-nc-nd/4.0/legalcode).

Pengo et al. observed a strong association between cortical pathology in RRMS and retinal microglial proliferation on OCT [19]. The authors found that hyperreflective foci, which represent activated and proliferating retinal microglia, were significantly higher in patients with RRMS compared to healthy controls (Fig. 3) [19]. In a similar study that utilized spectral domain OCT (SD-OCT) data, peripapillary retinal nerve fibre layer (pRNFL) and macular ganglion cell layer and inner plexiform layer (GCIPL) demonstrated significant atrophy [13]. GCIPL was also found to be a useful predictor of disability accumulation in early relapsing MS [20]. A study assessing macular volume, indicative of retinal ganglion cell neuronal integrity, revealed a significant 11% reduction in the eyes of individuals with a history of optic neuritis compared to control eyes (*P* < 0.001). Additionally, within the same patients, the affected eye exhibited a 9% decrease in macular volume compared to the unaffected eye (P < 0.001) [7]. Although Bruch membrane opening-minimum rim width (BMO-MRW) and pRNFL thicknesses are both reduced and associated with visual function defects in MS, GCIPL thickness may be the strongest predictor of visual impairment, according to recent OCT studies. [14, 21–23] Coric et al. utilized a measurement called inter-eye percentage difference (IEPD) of atrophy as a dimensionless parameter to distinguish health controls and MS patients. It was similarly found that the IEPD of the GCIPL had greater diagnostic accuracy than the more variable RNFL [24].

OCT has also provided insight into differentiating between MS subtypes. GCIPL is reduced in all subtypes of MS, but has been found to be significantly more reduced in SPMS relative to RRMS. GCIPL was also found to be better correlated than RNFL thickness changes in the same study [25]. When looking at non-affected eyes of MS patients, RNFL atrophy has been found to be greater in SPMS patients relative to CIS and RRMS patients [26, 27]. Total macular volume may also be a parameter for differentiating subtypes as it has been found to be more reduced in SPMS and PPMS relative to RRMS eyes [27]. OCT may also be a useful tool for monitoring disease duration and progression. Some studies have found an inverse correlation between GCIPL thickness and disease duration [25, 28, 29], which may be a useful characteristic for monitoring the effectiveness of therapeutic interventions. For only RRMS patients, the rate of thinning may also be correlated to disease duration [30, 31].

OCT imaging measurements of the GCIPL have proven instrumental in identifying significant differences that elevate it to a robust predictor of disability accumulation in MS. Heightened reduction in GCIPL within the SPMS subgroup highlights its potential utility in distinguishing different MS courses. Additionally, GCIPL demonstrated a superior correlation with disability accumulation when compared to other OCT measures, emphasizing its sensitivity in capturing disease-related changes. Moving forward, future directions for the GCIPL biomarker involve further exploration and refinement of its potential applications in MS diagnostics and prognostics. Investigating its performance in large-scale longitudinal studies can enhance its reliability as a predictive tool. Moreover, understanding the dynamics of GCIPL changes over time in response to therapeutic interventions will be crucial for assessing its utility in treatment evaluation.

##### Choroid

OCT imaging of the retina has demonstrated the strong prognostic value of measuring GCIPL and pRNFL; however, analysing choroidal thickness may have a similar ability for detecting MS. In a swept-source OCT (SS-OCT) study, the outer macular ring of the choroid was found to be significantly reduced in MS patients relative to healthy controls. There was a mild tendency of thinning in the peripapillary area, but no significant differences were detected [22]. Subfoveal choroidal thickness has also been found to be reduced and associated with longer disease duration [32]. In contrast, one study did not find a difference in choroidal thickness relative to control eyes, which suggests that future studies may be necessary to determine the role of the choroid in MS [33]. A common medication used for MS treatment is fingolimod, which increases vascular permeability and can lead to macular oedema. Kal et al. 2016 found that fingolimod users had increased choroidal thicknesses in the normal control group compared to the thinned MS patients [34]. Therefore, by leveraging baseline values, OCT emerges as a pivotal tool for nuanced monitoring. It not only allows us to track a potential deceleration in choroidal degeneration but also captures the broader context of an overall reduction in choroidal thickness, serving as valuable indicators for assessing MS prognosis and evaluating the impact of fingolimod treatment over time.

Future directions for utilizing choroid OCT measurements in MS prognostication involve conducting larger-scale longitudinal studies to establish more definitive correlations between choroidal changes and disease progression. These studies should consider the inclusion of diverse MS subtypes and incorporate standardized measurement techniques to enhance consistency across research findings. Moreover, the exploration of the choroid’s role in response to specific MS treatments (e.g., fingolimod) could contribute to personalized therapeutic strategies. Advancements in OCT technology, such as increased resolution and imaging depth, are crucial for refining the precision of choroid measurements. Future research should focus on standardizing methodologies, expanding longitudinal studies, and leveraging technological improvements to establish choroidal OCT as a reliable prognostic factor in MS.

#### OCT-Angiography (OCTA)

OCT-Angiography (OCTA) is another novel technique that has noninvasively allowed clinicians to visualize retinal and choroidal microvasculature changes in MS patients by detecting the motion contrast of blood cells [35]. In a meta-analysis that integrated retinal and choroidal OCTA data, the superficial vascular complex (SVC) and peripapillary vessel densities were found to be significantly decreased in MS patients with or without optic neuritis relative to the control group [36]. When combined with OCT-derived metrics, OCTA data was able to improve the detection of MS without optic neuritis [8]. Due to the retina’s structural and anatomic similarity to the brain, it is believed that the specialized tissue’s vascular changes reflect the cerebral vascular changes [11]. Evidently, the large effect size, consistency, and robust differences between MS patients and control eyes support the use of retinal oculomics in neurodegenerative diseases.

OCTA in the context of MS still warrants extensive investigations to solidify its role as a noninvasive biomarker for diagnosis and disease progression. Large-scale prospective studies are crucial to validate the reliability and specificity of OCTA findings, considering its potential application in diverse MS subtypes. Refining methodologies and establishing standardized protocols will enhance the consistency and comparability of results across studies. Exploring the utility of OCTA in tracking treatment responses and correlating microvascular changes with clinical outcomes can further broaden its clinical applicability. Additionally, longitudinal studies should focus on understanding the temporal dynamics of retinal and choroidal microvasculature changes, thereby advancing OCTA as a valuable and versatile tool in the comprehensive management and understanding of MS.

#### Machine learning and artificial intelligence

Integrating machine learning with oculomics and imaging techniques (e.g., OCT and OCTA) within the context of MS holds promise in revolutionizing neurodegenerative disease diagnosis and treatment modalities. Machine learning facilitates the analysis of extensive retinal imaging datasets, unveiling novel pathological patterns for efficient diagnosis. As a result, there will be enhanced predictive capabilities and earlier detection through less-invasive measures. Oculomics, as highlighted by Denniston et al. in their work on building trust in real-world data, may extend beyond biomarkers, emphasizing the broader implications of trust and reliability in utilizing health data for eye health and oculomics. Beyond data analysis, artificial intelligence collaborates with preclinical and clinical domains to establish consistency, fidelity, and clinical relevance through identified biomarkers. Maintaining such momentum of oculomics in the MS context will be crucial to realize its full potential and continue the advancement of diagnostic and treatment modalities of neurodegenerative diseases.

#### Fundoscopy

Fundoscopy (ophthalmoscopy) is a non-invasive technique that allows physicians to examine structures of the posterior segment of the eye (e.g., retina, optic disc, and retinal microvasculature). Optic neuritis is oftentimes one of the most common and often initial presentations of MS, developing in approximately 50% of MS patients [37]. MS optic neuritis patients will often experience visual problems, periorbital pain, and colour vision deficits [34]. On fundoscopy, characteristic presentation of optic neuritis may reveal papillitis and a hyperaemic optic disc with blurred margins. Early studies utilizing colour fundus photography and fundus fluorescein angiography have also been able to identify mild focal cuffing and retinal venous sheathing in MS patients [38].

Despite the effectiveness of fundoscopy as a primary method for detecting optic neuritis in MS, recent technological advancements in retinal diagnostic techniques, including scanning laser ophthalmoscopy (SLO), OCT, and visual evoked potentials (VEPs), have unveiled MS-specific characteristics with unprecedented precision. These cutting-edge tools not only enable early detection of retinal changes but also surpass the temporal limitations associated with symptom manifestation, allowing for a more comprehensive and proactive approach to the identification of MS-related alterations in the retina [39].

#### Scanning Laser Ophthalmoscopy

SLO is an advanced imaging technique that has emerged as a potential tool for observing alterations in RNFL thickness by detecting phase shifts when polarized light passes through birefringent structures [40]. Recent studies have demonstrated value in detecting ganglion cell loss in MS patients since demyelinating optic neuritis is a common characteristic [41]. The combination of adaptive optics and scanning laser ophthalmoscopy (AOSLO) has been able to further detect reduced cone reflectance, which suggests that cone composition changes in MS. AOSLO has also been able to visualize microscopic inner retinal vasculature changes around fundus vessels in an MS patient, however, future studies are warranted [42]. On ultra-widefield SLO (UWF-SLO), MS patients have been found to have thinner retinal arteries and veins relative to healthy volunteers, with significantly thinner arterioles in the nasal inferior quadrant. Microcystic macular oedema and peripheral retinal blood vessels in MS patients can also be analysed with SLO images if OCT is unavailable [43, 44]. Although SLO studies demonstrate promise for early diagnosis of MS, the defined parameters with OCT technologies may have more prognostic value.

Despite the promise of SLO in early MS diagnosis, future indications should consider refining parameters and exploring its prognostic value, particularly in comparison to well-defined parameters with OCT technologies. Prospective longitudinal studies are warranted to determine the temporal evolution of SLO-identified retinal alterations and their correlation with disease progression, providing critical insights into its prognostic potential. Additionally, investigating the feasibility of SLO in large-scale population studies and its utility in tracking treatment responses will contribute to establishing SLO as a versatile tool for both diagnosis and prognosis in MS. Advances in artificial intelligence applications for image analysis may further enhance the efficiency and accuracy of SLO, paving the way for its integration into routine clinical practice for MS management.

#### Tear biomarkers

The lacrimal gland is innervated by the lacrimal nerve from the cranial nerve (CN) 5, and the greater petrosal nerve (to lacrimal nerve) from CN 7, which supplies general visceral parasympathetic innervation. Tears are produced in the lacrimal gland, and subsequently empty into the lacrimal ducts and accumulate in the lacrimal lake before emptying out the puncta in the medial corner of the eye [45]. Tears function to deliver and excrete nutrients and products from the corneal epithelium and anterior stroma, thereby keeping the eye lubricated and moist. Proper tear production has also been shown to improve vision [46]. MS has the potential to lead to dry eyes through disruption of the lacrimal gland function and impairment of tear production. Ocular surface disease may be a manifestation of this pathophysiology [47]; however, tear composition may be of greater interest in the setting of MS screening.

Tear composition has been known to be affected by various disease states. For example, Tay-Sachs disease is associated with high levels of glycosidases in lacrimal gland secretions [48]. Even regular physiological changes can change tear composition; aging is associated with a decline in tear volume, flow, osmolarity, and film [47]. Research has started identifying biomarkers for MS in tears. One study used combined proteomics to identify elevated alpha-1 antichymotrypsin levels in tears of MS patients [49]. Since this marker was also found to be elevated in the CSF of MS patients, this could potentially serve as a less invasive biomarker diagnostic in place of spinal tap. However, Salvisberg et al. acknowledge that alpha-1 antichymotrypsin is also found in various other inflammatory disorders as well, warranting further investigation on the specificity of the biomarker [49]. Moreover, Hummert et al. found that oligoclonal band elevation in tears is not a specific biomarker for MS, while it is both a predictive and diagnostic biomarker when isolated from CSF [50].

Lipids and extracellular vesicles have also shown potential as MS biomarkers in tears, but further characterization is necessary [51]. Belviranli et al. indicated changes in tear quantity and quality in patients with MS. MS patients had higher conjunctival impression cytology grades and higher ocular surface disease index scores, while lower tear break-up time grades and Schirmer test scores [52]. Cicalini et al. indicated specific lipid matterns in MS patients with 15 phosphatidylcholine, 6 lysophosphatidylcholine and 11 sphingomyelin being down-regulated [53]. Higher levels of serine, histidine, aspartic acid, malonylcarnitine and/or 3 -hydroxy –valerylcarnitine, octenoylcarnitine and decenoylcarnitine, while lower levels of dodecenoylcarnitine, tetradecenoylcarnitine and 3-hydroxy-octadecenoylcarnitine were found in MS patients [54]. Evidently, a metabo-lipidomics analysis of tears in MS patients demonstrates prognostic value, but significantly more research is necessary before considering it as a valid screening method.

While studies have identified potential biomarkers (e.g., elevated alpha-1 antichymotrypsin levels, oligoclonal bands, lipids, and extracellular vesicles in tears) further investigation is crucial to establish their specificity and reliability for MS screening. The potential of alpha-1 antichymotrypsin as a less invasive diagnostic biomarker warrants in-depth exploration, given its presence in various inflammatory disorders. Additionally, the characterization of tear lipids and extracellular vesicles requires further scrutiny to delineate their role as MS biomarkers. The observed changes in tear quantity and quality In MS patients, including altered conjunctival impression cytology grades and ocular surface disease index scores, necessitate longitudinal studies to validate their consistency and applicability in clinical settings. Metabo-lipidomics analysis holds promise for prognostic value, but its integration as a screening method requires extensive research to establish its validity and specificity for MS detection. Future investigations should aim to refine tear biomarker profiles, explore their correlation with disease progression, and assess their potential as noninvasive diagnostic tools for MS.

#### Cornea

Corneal changes have been investigated as a potential diagnostic tool for MS due to ease of accessibility and relatively non-invasive methods of data acquisition. Ornek et al. found increased corneal sensitivity and tear function in neurodegenerative diseases such as MS [55]. Conversely, another study concluded that corneal sensitivity was not a useful differentiator, with the exception of only PPMS patients showing reduced corneal sensitivity. The other subtypes indicated no difference in corneal sensitivity [56]. Corneal nerve fibre density and nerve branch density have been found to be reduced in MS patients when utilizing corneal confocal microscopy [56]. The loss in corneal nerve fibres is associated with worsening neurological disability [57]. Total corneal immune cell density was higher in patients with MS (both RRMS and SPMS, but not clinically isolated syndrome (CIS)), however *immature* immune cell density was only higher in patients with MS and RRMS [58]. Possibly more importantly, MS, RRMS, and SPMS all had increased immune cell near-nerve distance compared to healthy controls. Khan et al. defined the near-nerve distance as the “nearest perpendicular distance between the IC cell body and nearest nerve,” which tended to increase with the loss of nerves.

Current studies provide glimpses into heightened corneal sensitivity and tear function in MS; however, the inconsistencies in differentiating subtypes warrant a more focused investigation. Advanced techniques such as corneal confocal microscopy offer the potential to delve into corneal nerve fibre density and immune cell characteristics, providing valuable insights into the pathophysiology of MS. However, to establish corneal changes as a consistent and reliable screening method, future research should concentrate on standardizing parameters, understanding variations in immune responses across MS subtypes, and defining the dynamics of immune cell near-nerve distance. This thorough exploration will contribute to the development of a robust and clinically applicable corneal biomarker profile, fostering its potential integration into routine MS screening with confidence and specificity.

#### Pupil

Cognitive fatigue, a symptom of MS, is associated with a reduced pupil response time in MS patients, especially those in the high cognitive loss load condition [59]. This finding was corroborated by Meltzer et al. who found MS patients with “significant attenuation of the melanopsin-mediated sustained pupillary response.” [60] This response seems to be reduced regardless of the time of stimuli. Patients with MS have also been found to have reduced multifocal objective pupil perimetry, with delayed time to peak [61]. In addition to reduced pupil contraction amplitude, RRMS patients had reduced initial pupil diameter [62].

The association between reduced pupil response time and cognitive fatigue in MS patients, particularly in high cognitive load conditions, suggests a potential avenue for assessing cognitive aspects of the disease. Further research should delve into the mechanisms underlying the melanopsin-mediated sustained pupillary response, exploring its consistency over time and its correlation with disease progression. While pupil oculomics alone may not serve as a standalone diagnostic tool for MS, future considerations should focus on its integration with other established gold-standard methods.

#### Visual evoked potentials

Electrical signals called visual evoked potentials (VEPs) are produced by the visual system in response to visual stimuli (i.e., evoked potentials) such as a flashing light (flash VEP) or checkerboard pattern (pattern VEP). The photoreceptor cells in the retina convert the light energy from a visual stimulus into electrical signals where they are subsequently carried by the optic nerve to the visual cortex. The function of the optic nerve and the visual cortex, which are frequently impacted by diseases such as MS, optic neuritis, and glaucoma, can be evaluated using VEPs. Clinicians can detect anomalies in the visual system and track the course of the disease by analysing the latency (time delay) and amplitude (intensity) of the VEPs. VEP latency was found to have prognostic value in predicting futural disability of MS when analysing clinically unaffected eyes [63, 64]. When used in conjunction with MRI data, prolonged VEP latency was significantly associated with reduced whole brain volume, grey matter volume, and white matter volume [65], providing evidence of prognostic potential for MS [9]. VEP abnormalities have even been demonstrated to have higher sensitivity for detecting MS lesions of the visual pathway than OCT [66].

The ability of VEPs to assess the function of the optic nerve and visual cortex provides a valuable diagnostic tool for MS. Research should focus on improving the precision and reliability of VEP analysis, exploring the potential correlations between VEP abnormalities and specific characteristics of MS lesions. Furthermore, investigating the prognostic value of VEP latency in clinically unaffected eyes could contribute to the early prediction of future disability in MS patients. Integrating VEP data with advanced imaging techniques such as MRI offers a multidimensional approach, enhancing the understanding of disease progression. Future studies should aim to establish standardized protocols for VEP assessments, ensuring consistent and accurate measurements that can be reliably utilized for diagnostic and prognostic purposes in MS.

#### Functional and oculomotor visual markers

##### Visual acuity and visual sensitivity

Low contrast letter acuity (LCLA) is emerging as the leading metric in measuring visual disability in patients with MS [5]. While there is no discernible difference in high contrast visual acuity, there is a marked decrease in LCLA in MS patients compared to disease-free controls [67, 68]. LCLA deficits in MS manifest as decreased contrast sensitivity due to damage to a specific inter-neural connections in the visual pathway. These injuries severely impact integral activities of the patient’s day-to-day functioning such as reading, driving, and facial recognition [69–71]. In addition to reduced visual acuity, contrast sensitivity is another visual dysfunction that manifests in MS. Contrast sensitivity is the product of ganglion cell layer structural defects, which results as inability to perceive sharp outlines of objects [72]. Deficiencies in LCLA and contrast sensitivity can additionally be associated with reduced retinal nerve fibre layer (RNFL) thickness [72, 73]. While visual acuity and contrast sensitivity may serve as a rapid assessment for evaluating MS progression or treatment efficacy, they lack specificity. Therefore, visual acuity and sensitivity assessments should be used in conjunction with other biomarkers to properly diagnose MS and the severity of the disease.

##### Stereopsis and depth perception

Depth perception may be significantly impaired in MS patients due to lesions in Brodmann Areas 17 and 18. Area 17 of the primary visual cortex is primarily involved with comprehending depth perception, while Area 18 of the visual association cortex is responsible for spatial organization [74, 75]. Together, these serotoninergic innervated neurons modulate and process ascending visual information. In MS, these areas have reduced serotonin concentrations which may be the causative factor resulting in depth perception deficits [76]. An area of currently conducted therapeutic research is the use of transcranial electric stimulation which may stimulate serotonergic transmission in Areas 17 and 18, reducing the severity of the symptoms [77–79]. Similar to LCLA, stereotests may be used to assess MS progression or treatment efficacy, but depth perception alone lacks specificity to be used as a biomarker for MS.

##### Ocular Motility and Saccadic eye movements

Internuclear ophthalmoparesis (INO) is the most common saccadic disorder observed in patients with MS. INO is caused by demyelination and subsequent diminished signal transmission through the medial longitudinal fasciculus (MLF) in the medial tegmentum of the pons. Binocular coordination (conjugate eye movement) is aberrantly disrupted due to slowing of the adducting eye during horizontal saccadic movements [80]. This has been described as an adduction lag and is most prominent during the fast phases of optokinetic reflex testing. Clinically, this presents as dissociated nystagmus of the abducted eye and is differentiated from true nystagmus because it is comprised of saccadic oscillations [81].

MS patients with INO will typically present with diplopia, blurred vision, and visual confusion when undertaking tasks that require binocular fusion [80]. INO often presents unilaterally but can also present bilaterally. Unilateral INO may have components of vertical diplopia whereas in bilateral INO, additional smooth eye movements will be impacted due to impairment of the vertical vestibulo-ocular reflex and smooth pursuit signals from vestibular nuclei. This is because the axons of the MLF carry vestibular and smooth pursuit signals from the vestibular nuclei to the midbrain nuclei to facilitate vertical gaze [82]. INO can be quantified and used as a metric to measure disease severity by evaluating the peak velocity of the abducting eye versus the adducting eye during saccades with infrared oculography [83], scleral search coil, or video oculography-based techniques [84]. Studies have consistently shown an increase in peak velocity in patients with INO compared to normal controls [85, 86]. Objective measures of peak velocity may be used to suggest clinical severity of chronic MS and potentially correlate to neuroradiological abnormalities in the MLF [86].

The second most common saccadic disorder after INO is saccadic dysmetria, caused by lesions in the cerebellar peduncles [87]. Lesions in the flocculus and paraflocculus, which comprise the vestibulo-cerebellum, can manifest as impaired smooth pursuit and inability to suppress the horizontal vestibulo-ocular reflex (VOR) during tasks that involve combined eye-head tracking [88]. Gaze-evoked nystagmus (GEN) and downbeat nystagmus (DBN) are other types of saccadic dysmetria that are associated with a defect in the neural integrator network and loss of inhibitory cerebellar control on the vertical semicircular canals, respectively [89, 90].

Evidently, tracking of dysmetria and saccadic eye movements demonstrates prognostic potential for evaluating the severity of MS as well as the therapeutic effect of various treatments [91]. In the recent rapid development of virtual reality (VR), stronger eye tracking technology in VR platforms holds promise for capturing and analysing precise eye movements, allowing more detailed assessment of oculomotor abnormalities in MS [92]. Paired with machine learning, researchers may enhance the diagnostic accuracy of saccadic eye movements with VR and open new insights on the functional and oculomotor visual markers throughout disease progression.

## Conclusion

The collaboration between oculomics and current diagnostic techniques holds promise for enhancing the prognostic value of identifying and monitoring MS. There is clearly a need for more accurate and earlier detection of MS; however, the gold standards for diagnosis, such as MRI and CSF spinal taps, are expensive and invasive, making population-wide screening challenging. By leveraging ocular biomarkers found in the tears, cornea, pupil, and especially the retina, future revisions to the McDonald criteria may improve the accuracy and timeliness of MS diagnosis. Functional biomarkers, such as eye movements, visual acuity, and sensitivity, may provide additional screening modalities to supplement biological and neuroimaging techniques (Table 1).

**Table 1:** Ocular structures and their associated pathological changes in Multiple Sclerosis.

Ocular and Functional Biomarker	Diagnostic Modality and Key Findings
**Tears**	• Elevated levels of alpha-1 antichymotrypsin [49] are detected in the tears of Multiple Sclerosis (MS) patients, accompanied by higher grades of conjunctival impression cytology, increased ocular surface disease index scores, and lower tear break-up time grades and Schirmer test scores [52, 53].
**Cornea**	• Corneal sensitivity and tear function decline in neurodegenerative diseases, evidenced by reduced corneal nerve fibre and nerve branch density in MS patients [55, 56]. Additionally, higher total corneal immune cell density, particularly in patients with MS and RRMS [58], alongside increased immune cell near-nerve distance, distinguishes MS subtypes from healthy controls [58].
**Pupil**	• Cognitive fatigue is linked to a shortened pupil response time, particularly evident in MS patients under high cognitive load conditions [59, 61].
**Retina**	**Fundoscopy** • Optic neuritis frequently emerges as an early indicator of MS, manifesting in around 50% patients [37, 38]. **Scanning Laser Ophthalmoscopy (SLO)** • MS patients show thinner retinal arteries and veins, notably in the nasal inferior quadrant [43], and SLO images are useful for analysing microcystic macular oedema and peripheral retinal blood vessels when OCT is unavailable [42]. **Optical Coherence Tomography (OCT)** ***Retinal Nerve Fibre Layer*** • RNFL thinning is prominent in MS, with affected eyes displaying a substantial average reduction of 46% (P < 0.01), and a specific 28% reduction when compared to unaffected eyes. Additionally, when comparing unaffected eyes to control eyes, there is an average RNFL reduction of 26% (P < 0.01) [26, 27]. Peripapillary retinal nerve fibre layer (pRNFL) and macular ganglion cell layer, and inner plexiform layer (GCIPL) also demonstrated significant atrophy [13]. ***Ganglion Cell and Inner Plexiform Layer (GCIPL)*** • GCIPL is consistently reduced across all MS subtypes, with a notably more significant reduction in secondary progressive MS (SPMS) relative to relapsing-remitting MS (RRMS) [24, 25]. Total macular volume also emerges as a differentiating parameter, being more reduced in SPMS and primary progressive MS (PPMS) relative to RRMS eyes [27]. GCIPL is identified as a valuable predictor of visual impairment and disability accumulation in early relapsing MS. [14, 20–23] **Optical Coherence Tomography-Angiography (OCTA)** In MS patients, both with or without optic neuritis, there is a significant decrease in the superficial vascular complex (SVC) and peripapillary vessel densities compared to the control group [36]. **VEP** • VEP latency was significantly associated with reduced whole brain volume, grey matter volume, and white matter volume [65]
**Choroid**	**Swept-source OCT (SS-OCT)** • The outer macular ring of the choroid was found to be significantly reduced in MS patients relative to healthy controls [22]. **OCT** • Subfoveal choroidal thickness is diminished in correlation with prolonged disease duration [32]. However, findings by Masala et al. in 2022 did not reveal a significant difference in choroidal thickness compared to control eyes [33].
**Visual acuity and sensitivity**	**Low contrast letter acuity (LCLA)** • A significant reduction in LCLA is evident in MS patients compared to disease-free controls [68, 69], and deficiencies in LCLA and contrast sensitivity are linked to diminished retinal nerve fibre layer (RNFL) thickness [73, 74].
**Stereopsis/Depth Perception**	• Depth perception may be significantly impaired in MS patients due to lesions in Brodmann Areas 17 and 18 [80]
**Eye Movements**	• Patients with internuclear ophthalmoplegia (INO) demonstrate increased peak velocity compared to normal controls [86, 87]. The disparity in peak velocity between abducting and adducting eyes during saccades can be measured using infrared oculography, scleral search coil, or video oculography-based techniques [85].

Future research should delve deeper into the complexities of each subtype of MS to identify specific ocular biomarkers associated with disease progression and therapy responsiveness. These contributions would lay foundations based more on a comprehensive understanding of the heterogeneity within MS and assist in developing more targeted interventions. In the advent of more powerful machine learning algorithms, screening modalities such as OCT, eye tracking, and protein analysis become more effective tools aiding in MS diagnosis. AI can analyse larger and more diverse data sets to potentially discover new parameters of pathology for efficiently diagnosing MS before symptom onset. These developments will translate into earlier detection for facilitating the timely initiation of DMTs, which would slow disease progression and enhance the quality of life for patients.
